# A Pilot Study on the Freelisting Method Among Adolescents with Chronic Musculoskeletal Pain: Feasibility, Acceptability and Study Findings

**DOI:** 10.3390/children12010048

**Published:** 2024-12-31

**Authors:** Sabrina Gmuca, Dori Abel, Mackenzie McGill, Daneka Stryker, Eda Akpek, Whitney Eriksen, Pamela F. Weiss, Peter F. Cronholm

**Affiliations:** 1 Department of Pediatrics, Division of Rheumatology, Children’s Hospital of Philadelphia, Philadelphia, PA 19104, USAmcgillm1@chop.edu (M.M.); weisspa@chop.edu (P.F.W.); 2 Center for Pediatric Clinical Effectiveness, Children’s Hospital of Philadelphia, Philadelphia, PA 19104, USA; 3 PolicyLab, Children’s Hospital of Philadelphia, Philadelphia, PA 19104, USA; peter.cronholm@pennmedicine.upenn.edu; 4 Perelman School of Medicine, University of Pennsylvania, Philadelphia, PA 19104, USA; 5 Drexel University College of Medicine, Drexel University, Philadelphia, PA 19129, USA; dstryker@chla.usc.edu; 6 Mixed Methods Research Lab, Perelman School of Medicine, University of Pennsylvania, Philadelphia, PA 19104, USA; 7 Center for Public Health, Department of Family Medicine and Community Health, Leonard Davis Institute of Health Economics, University of Pennsylvania, Philadelphia, PA 19104, USA

**Keywords:** freelisting, resilience, chronic musculoskeletal pain, cognitive behavioral therapy, psychology, adolescence

## Abstract

**Background/Objectives**: To assess the feasibility and acceptability of freelisting for adolescents with chronic musculoskeletal pain (CMP) and use freelisting to identify how adolescents with CMP cope with pain. **Methods**: This was a mixed-methods cross-sectional single-center study of patients 12–18 years old, diagnosed with CMP. Twenty-seven subjects participated in an interview which included the freelisting exercise, probing questions, Connor-Davidson Resilience Scale 10-item, and semi-structured interview. Feasibility was pre-defined as ≥85% completion. A list of ‘standardized’ freelisting terms was created, and we calculated Smith’s salience index. Freelisting terms were grouped into individual, family, friends, school, and medical domains. **Results**: In this predominantly female population, resilience levels were low to moderate, and on average, participants had moderate pain intensity and functional disability. The freelisting exercise was feasible and acceptable among females, with low recruitment of males. Salient words to describe adolescents’ pain included frustrating, upsetting, annoying, and painful. Participants identified family, friends, teachers, guidance counselors, and health professionals as key sources of support. Many participants reported difficulties communicating their pain. Helpful coping strategies included rest, distraction, relaxation, endurance, and extracurricular participation. **Conclusions**: Freelisting was well perceived among female adolescents with CMP. Supportive relationships with community members play an important role for this population. However, perceived stigma may deter female adolescents from talking about their pain. Purposive sampling of male participants and recruitment strategies ensuring diverse patient representation are necessary to ensure generalizability of future results when using the freelisting method for adolescents with CMP.

## 1. Introduction

Chronic musculoskeletal pain (CMP) is the third most common cause of chronic pain among adolescents, affecting approximately 20% of children and adolescents [1,2]. Chronic pain during adolescence presents several challenges including the stress of managing multiple treatment components, which may include adhering to a difficult, multidisciplinary non-pharmacologic treatment regimen [3,4]. Adolescents with CMP who adopt physical and psychological adaptive coping strategies, despite their pain, can be considered resilient [5]. Our team previously demonstrated that adolescents with CMP who have greater levels of self-perceived resilience have lower levels of symptomatology and higher levels of health-related quality of life compared to their less resilient peers [6,7]. Incorporation of patient perspectives regarding the coping strategies and resilience resources that work best for adolescents with CMP has the potential to inform the enhancement of existing models and build new approaches. Similarly, a better understanding of the coping skills that adolescents with CMP commonly access and find helpful would provide targets to guide further tailoring of psychological-based interventions for this vulnerable patient population.

Because pain is a subjective phenomenon and affects multiple domains of life, including the patient voice in clinical research should be considered paramount and prioritized in the pain research community [8]. One method that can be used to investigate the patient perspective is freelisting: a standard anthropological technique used in Cultural Consensus Analysis for gathering data about a specific topic or domain by asking individuals to list all the items they can think of that relate to the presented topic [9,10]. Freelisting is effective for learning about the knowledge, attitudes, and practices that are common and important within a group or culture conceptualized as the unit of analysis [11]. The ease and truth of freelisting makes it ideal for collecting data on health information and theories from fairly large samples [11]. In freelisting, the researcher prompts participants to create brief, open-ended, often one-worded lists in response to the topic of interest [11]. Freelisting produces a measure of salience for generated responses, providing content areas of focus as well as exemplar language used within the respondent group [12]. Freelisting has recently grown in popularity and has become an excellent tool for rapidly exploring how groups of people think about and define a particular health-related domain [12,13,14]. However, there is limited published use of freelisting among adolescents, and none, to our knowledge, among adolescents with CMP [15,16,17,18,19,20]. The study’s objectives were to a) assess the feasibility and acceptability of freelisting for adolescents with CMP and b) use freelisting to identify how adolescents with CMP, established in a pediatric rheumatology subspeciality chronic pain clinic, cope with pain.

## 2. Materials and Methods

### 2.1. Study Design, Population and Setting

This was a nested mixed-methods cross-sectional study. The study was conducted between December 2020 and January 2022 at one investigative site: The Children’s Hospital of Philadelphia. Participants were English-speaking patients 12–18 years old, diagnosed with CMP (including amplified musculoskeletal pain syndrome and chronic widespread pain) by the treating physician and seen for a follow-up visit in a pediatric rheumatology subspecialty pain clinic. Exclusion criteria included: (1) presence of a co-morbid inflammatory (e.g., juvenile idiopathic arthritis, systemic lupus erythematosus, etc.), neurologic (e.g., complex regional pain syndrome, neuropathies), or oncologic/hematologic condition (e.g., sickle cell disease, cancers), (2) pain limited to the abdomen or head, (3) functional neurologic symptom disorder and (4) cognitive impairments that would preclude participation in the study. This study received local institutional review board approval (IRB 20-017961). The two interviewers underwent training for conducting interviews. In this training, best practices for interview facilitation as well as practice with the interview guide for this study were completed to limit bias in the interview process.

### 2.2. Recruitment

We set a goal sample size of 30 participants, as this is well above the number demonstrated as adequate in qualitative research to achieve saturation (9–17 interviews) [21,22,23]. Potential participants were contacted via telephone prior to their scheduled follow-up clinic visit. The format and purpose of the study was explained to gauge interest. Patients and parents meeting eligibility criteria were consented (assented if under 18 years of age) at the time of the clinic visit or later over the telephone via verbal consent. Participants were compensated with a USD 20 e-gift card for their time and completion of study procedures.

### 2.3. Study Procedures

Study respondents participated in one interview session which was held via telephone or video conferencing, based on respondent preferences. The interview was comprised of the following four parts:

Freelisting exercise. Participants were asked 12 questions about coping activities for their CMP, sources of support, and words they use to describe their CMP among various settings. These questions were informed by existing models of psychological resilience and the Consolidated Framework for Implementation Science Research (CFIR) [7,24,25,26]. These included individual, family, social and cultural domains. Participants responded to each question using as many one-word or short answers they could list. The freelisting prompts were developed and an initial trial of three interviews were conducted with patients to assess structural flow and feasibility.

Probing questions. Participants were asked to clarify the significance of one word from each freelist they produced. The freelist words were chosen at the discretion of the interviewer. The interviewer could choose any word to help clarify unique or unclear responses or, if there was no obvious choice, the first word in the freelist was used.

Connor-Davidson Resilience Scale 10-item (CD-RISC-10) [27]. Participants verbally completed this 10-item questionnaire that measures overall self-perceived resilience, or ability to cope with stressors/challenges. Higher scores (on a scale of 0–40) indicate greater resilience. It has good internal consistency and test-retest reliability and can be used to assess treatment response [27].

Semi-Structured Interview. Respondents participated in semi-structured interviews across six categories to elicit participants’ feedback regarding the freelisting exercise. Questions addressed feasibility, acceptability, and suggestions for adaptations or improvements of the freelisting exercise. The interview was guided by The Consolidated Framework for Implementation Research (CFIR), which is a well-operationalized and widely used framework to assess potential barriers and facilitators within local settings [26]. Questions addressed freelisting content, timing, duration, delivery, attendance, and overall satisfaction to investigate the likelihood of uptake by other researchers and ways to improve it.

### 2.4. Data Collection

Audio recordings from the interviews were transcribed by a HIPAA-compliant external vendor and cleaned of identifying information. Patient demographics and clinical characteristics were abstracted from the electronic medical record in Research Electronic Data Capture (RedCap 14.8.3) and merged with data from the clinic’s existing patient registry, which captures patient-reported outcome measures and detailed pain-specific medical history. This included: gender, race, ethnicity, age, pain subtype, pain visual analog scale (VAS), functional disability inventory (FDI) self-report [28], widespread pain index (WPI) [29], symptom severity score (SSS) [29], and the PROMIS (Patient-Reported Outcome Measures Information System) [30] measures for fatigue (PROMIS pediatric short form V1.0-Fatigue) and peer relationships (PROMIS PEDIATRIC short form v1.0-Peer relationships), and the child version of the pain catastrophizing scale (PCS-C) self-report [31].

### 2.5. Data Analysis

#### 2.5.1. Quantitative Data

Feasibility of the freelisting method was pre-defined as ≥85% of participants completing the process. We used 85% as the cut off for feasibility, given that the study “intervention” (participation in the freelisting method), was deemed to be much less onerous than participation in a resilience coaching program for which we previously demonstrated a completion rate of 81% for a similar patient population [32]. The goal enrollment rate was defined as ≥30% based on our enrollment rates from previous studies in this patient population ranging from 25–70%. Baseline and demographic characteristics were summarized by standard descriptive summaries using non-parametric and parametric statistics, as appropriate. We reported summary statistics for CD-RISC-10 scores.

#### 2.5.2. Qualitative Data

To identify salient freelisting terms, our study team worked with the University of Pennsylvania’s Mixed Methods Research Lab (MMRL). The research team comprised individuals with diverse but relevant expertise including clinical rheumatology, chronic pain, qualitative research methodology, and patient centered care. Members brought varying levels of pre-knowledge regarding CMP based on their professional roles and prior experiences.

- Clinical expertise: The principal investigator, a pediatric rheumatologist, had extensive experience treating patients with CMP, which provided clinical insights into the complexity of symptoms and their impact on patients’ daily activities.
- Qualitative research experience: Team members with qualitative research training contributed expertise in data collection and analysis methods, ensuring a rigorous and unbiased approach to exploring patient perspectives.
- Personal and indirect exposure: Some team members had indirect exposure to CMP through prior work in related research fields, providing an understanding of its biopsychosocial domains.

To minimize potential biases stemming from pre-knowledge, the team engaged in reflexivity exercises, such as discussing individual assumptions and expectations about the study topic prior to data collection. Regular team meetings ensured ongoing awareness of how pre-knowledge might shape interpretations of the data. Furthermore, investigator triangulation was implemented by involving five researchers in data coding and interpretation.

We extracted participant responses to the freelisting questions and reviewed terms across participants and questions to create a list of ‘standardized’ terms. The standardization process involved several meetings to consolidate synonymous terms, phrases, and grammatical variants (plurals and gerunds). The team reviewed the items in pairs. Discrepancies were identified and resolved by consensus. The final lists were formatted in Microsoft Excel and imported into Anthropac v 4.98 for cultural consensus analysis. Anthropac utilizes standardized algorithms to develop salience indices (Smith’s salience index [S]) for each item identified for each freelisting question [9,33]. Smith’s salience index represents both the frequency of an item across all respondents’ lists as well as its rank order within lists. Scree plots based on sorted salience indices were created in Microsoft Excel and examined to identify items above the leveling off of each scree plot—i.e., the top salient items. To evaluate saturation, we analyzed the results of the freelisting exercise, focusing on comprehensiveness and redundancy of the responses across participants. Saturation was determined when no new, meaningful items or themes emerged in successive freelists. This process involved organizing the items elicited in the freelisting exercise, ranking them by frequency, and analyzing the cumulative frequency of mentioned items. We monitored for stabilization in the frequency and diversity of the items, indicating that additional participants were not contributing novel or substantively different responses. The point at which new freelists yielded negligible variations or additions to the data was considered evidence of saturation.

Semi-structured interviews were coded and analyzed using NVivo 13 (2020, R1). Two members of the study staff coded transcripts independently and met to identify emergent themes arising from a line-by-line review of narrative content as well as a priori codes. 20% of interviews were double coded to insure intercoder reliability of the developed coding schema. Discrepancies were resolved through consensus. Interviews were analyzed for thematic saturation (i.e., when successive interviews revealed no new themes). Saturation was reached with 30 completed interviews. An initial trial of 3 interviews was conducted with patients to assess structural flow and feasibility. Minor wording changes were then made to improve clarity. This process occurred between November 2021 and January 2022.

## 3. Results

Charts for 151 participants were screened, of whom 71 (40.8%) were eligible for this study. A total of 29 participants were enrolled (41.1% enrollment), and 27 (93.1%) completed all study procedures. Reasons for non-participation were not identified because uninterested participants did not respond to invitations for the study or did not follow-up with scheduling a telephone call after consenting. Table 1 provides patient demographics and clinical characteristics for participants. Participants were overwhelmingly female (96.3%). The majority of participants were White (77.8%) and non-Hispanic (88.9%). The average age was 16 (1.8) years, and the participants predominantly had diffuse pain (81.5%), affecting three or more body regions. Resilience levels were low to moderate, with a mean score on the CD-RISC-10 of 27.2 (6.4). On average, participants had moderate pain intensity and moderate functional disability [34]. Time between interview and most recent clinic follow-up visit was a mean of 13.9 (39.2) days, and time from initial clinic visit to interview date was a mean of 462.4 (364.8) days.

In the freelisting exercise portion, the recording for one participant was cut off, resulting in only half of their responses being captured for analysis. Therefore, for freelisting items 1–5, *n* = 26, and the remainder *n* = 27.

**Figure 1:**
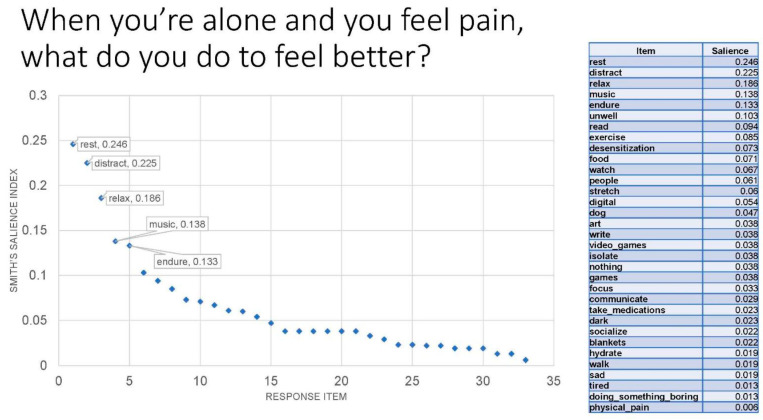
Scree Plot for Question #1: When you’re alone and you feel pain, what do you do to feel better?

Table 2 and Figure 1, respectively, display the item list in decreasing salience index rank order and the scree plot for Question #1. The item lists and scree plots for all other freelisting questions are available as Supplementary Material. Table 3 presents the 12 freelisting questions in order of the original freelisting exercise and the top salient items for each question. Freelisting terms were grouped into subject areas related to the individual self, family, friends, school setting, and medical setting. Quotes from participant’s answers to the probing questions were used to elucidate and clarify some of the salient words.

### 3.1. Individual Self

Participants described that when they’re alone and feel pain, they try to “rest”, “distract”, or “relax” themselves to alleviate pain symptoms and feelings surrounding pain. One salient strategy identified included “music”, such as listening to music or playing an instrument. Participants who listed “endure” or similar phrases, such as “tough it out” and “push through”, described tolerating pain so that they can resume their activities and not miss out on being around others because of their pain.

*“But a lot of times to distract, I try to do something that I enjoy. So, I read or I draw and do some photography. I like school, so sometimes I study if I can focus, but a lot of times when I’m in a lot of pain, I can’t focus on anything. So, if that’s the case, I talk to my friends, [or] I do something that’s enjoyable”.*

*(Participant #04)*

*“Like my psychotherapist, he [taught] me breathing techniques to calm myself down and…distract myself from the pain and the anxiety”.*

*(Participant #23)*

Participants used terms related to “frustrating” and “sad” to describe how they think or talk about their pain. Often, participants used these terms to describe their inability to change their chronic pain.

*“I think it’s just like, sometimes there’s not a lot I can do to make it go away, so it’s frustrating that there’s nothing I can do”.*

*(Participant #22)*

*“I’m never sad in the point where it’s so bad, but there’s always a little bit of sadness that, yeah, I have to go through this”.*

*(Participant #06)*

### 3.2. Family

Participants used words related to the salient terms “communicate” and “distract” to describe how they cope with their pain when with their families. Respondents detailed that they engage with family members to divert their attention away from pain symptoms as well as feelings about their pain and that they do not talk to their family members about their pain. Reasons why participants don’t talk to their family members about their pain varied greatly among individuals. Some explanations included wanting to avoid thinking about their pain to prevent symptom exacerbation, preferring to cope with pain alone, or feeling that the situation isn’t something that can be resolved by talking about it. Participants identified family members (specifically parents and siblings), along with friends, as salient support networks.

*“[Talking] distracts my mind. That’s what I really do to cope with my pain, is distract myself”.*

*(Participant #29)*

*“It’s not that they don’t care or anything like that, but there’s not really too much to talk about because they already know how it is. Like, they already know that it’s going to be there. They already know it’s going to be worse if I do physical movement and stuff like that, so they expect that. Sometimes, I’ll mention like, it feels worse a particular day, but not like in particular, we don’t really talk about it too often”.*

*(Participant #13)*

### 3.3. Friends

Participants identified friends as both salient support networks and community members helpful for their pain.

*“[My friends are] just there for me, no matter what I need… if I need someone to talk to or if I need someone to visit me or just whatever I need, they’re there to help”.*

*(Participant #16)*

The most salient strategy that participants use when they feel pain while with their friends is to “communicate” to get support coping with their diagnosis or to let their friends know they’re experiencing pain and need to rest or excuse themselves. One participant described how explaining their chronic pain condition helped their friends be better supports:

*“[I’ve] found that… [my friends] don’t need to know everything, but if they’re curious, explaining [what CMP is] will make them understand more, and that’ll make you feel more supported, that there are people who care about you, and who know what’s going on”.*

*(Participant #08)*

Other participants responded that they don’t discuss their CMP with their friends, to keep the details of their condition private and because CMP is a complex condition to explain.

*“I try not to bother them necessarily to get into too much detail. They don’t really know much about it, because well, kids my age don’t usually have this type of thing. So, I just don’t talk about it”.*

*(Participant #23)*

Among those who did talk about their CMP with their friends, some participants used words or phrases related to the medical condition itself, such as “AMPS [amplified musculoskeletal pain syndrome]”, “fibromyalgia”, and “EDS [Ehlers-Danlos syndrome]”, along with a simplified explanation of their pain condition. Other participants reported using the term “annoying” to describe their emotions surrounding pain when around their friends.

*“So, yeah, my friends understand even though they are confused about it, but they understand my side. I’ll say AMPS is just a nerve condition that I have and they’ll say okay. They already know I have pre-existing back issues and ankle issues so I just have to say, my AMPS”.*

*(Participant #20)*

*“… I would go like, ‘Oh, this is so annoying,’ because [my friends] wouldn’t understand if I go into detail, most of these like neurological like feelings and tingles… So, saying that you feel little pins and needles around your shoulder or that you don’t feel sensation somewhere, a lot of people don’t understand that. So, kind of just saying like, ‘Oh, it’s so annoying that I can’t feel something right here’, is a better way to describe it”.*

*(Participant #07)*

### 3.4. School Setting

Participants listed resting, distracting, relaxing, focusing (on the school lesson), and leaving (excusing themselves to take a small break) as methods for feeling better when they’re at school and experiencing pain.

*“… I’ll acknowledge the pain [is] there, but… if I’m in the middle of learning something, and I’m focusing so much on the pain, it’s really hard to focus in school, and I feel like I’m missing out on what I’m supposed to be learning. So I find it’s best for me to…try to not let it like affect too much of what I’m supposed to be doing in school”.*

*(Participant #11)*

*“[If] I can’t concentrate on the work… [I will] step away and kind of take a deep breath, and then go back into the class, because sometimes if I’m just sitting there with the pain, it’s kind of hard to focus on other things going on”.*

*(Participant #26)*

Participants identified teachers and guidance counselors as helpful community members for their pain, through their provision of accommodations and flexibility for completing coursework.

*“My guidance counselor has been really helpful to me [these] past two years when I was trying to like figure things out. She understands what I’m talking about, and so, if I say that I need a day off or like an unexcused absence for that, she’ll try to work with me and work with my teachers so my teachers can work with me to get through it. It’s really helpful to have somebody at school who is on my side”.*

*(Participant #13)*

The most salient term participants used to describe talking with teachers about their pain was “painful”, that is, participants telling their teacher that they’re in pain. One participant explained how some teachers did not understand their AMPS condition; therefore, they might offer a simplified explanation “that they’re in pain”, rather that describing the full details.

*“[Some] teachers were not able to understand or conceptualize what was happening [with my CMP]….Sometimes, it was like… [I would] explain what I had, or it was just like, ‘I have a painful reaction because I did this to it’.*

*(Participant #07)*

Participants described a range of extracurricular and group activities helpful for their pain, including performance arts (theater, singing, music), organized sports, other types of exercise (running, weightlifting, yoga), visual arts (drawing, coloring, painting), extracurricular clubs (such as student government and cultural clubs), and general opportunities to socialize with friends.

*“…I started playing the cello when I was in my fourth grade, [and] it’s something that’s always been a part of my life, something that’s very consistent, very grounding. Doing those kinds of things are helpful and distracting, but also doing something you enjoy doing, regardless of the pain, it gives you relief… When you’re busy doing something that you love, and something that comes naturally to you, it’s a nice way to kind of trick your body into not thinking about that pain”.*

*(Participant #08)*

### 3.5. Medical Setting

Participants identified doctors, physical therapists, and behavioral therapists as the most salient health professionals who are helpful for their pain. They appreciated having doctors and physical therapists who had expertise in treating CMP and cared about ameliorating their condition. Participants also described behavioral therapists as a beneficial resource for talking about their CMP condition and for helping to address stressors that might aggravate their symptoms.

*“[My doctor] has like an extensive past and experience with [CMP], even though he supposedly doesn’t have it. Like, he still understands because people are explaining their symptoms to him, like every day, 24/7… knowing that, what I say, he’s going to have heard before and he knows how it affects me and what to do with that, [is] important”.*

*(Participant #19)*

### 3.6. Feedback on Freelisting Exercise

Most participants reported that they liked the freelisting exercise, including its questions and overall format, and several participants shared that they liked the study’s goals, notably the idea of helping others build resiliency related to their CMP. Some participants desired additional questions that would allow them to share more nuanced information about their pain-related symptoms, the severity of their CMP, and its effect on their mental health, emotional wellbeing, and everyday choices.

*“I mean, I know it’s like a pain disorder, but a lot of patients with it have like other symptoms than just pain. Like my AMPS causes me to pass out and get migraines and throw up and stuff like that. So, maybe more questions about the other symptoms that comes with AMPS and stuff”.*

*(Participant #02)*

*“[So,] you asked me what I do when I’m with my friends, but a lot of times in the height of AMPS, I’m not with my friends…so [maybe ask about] how AMPS dictates what you do or doesn’t or…what role it plays in your life…”*

*(Participant #04)*

## 4. Discussion

The freelisting exercise used in this study was feasible and well-accepted among study participants and offered a unique avenue for sharing their pain-coping strategies. However, all but 1 participant was female. While patients were recruited for this study regardless of biological sex, the study sample skewed female. While this may be reflective, in part, of the overall female predominance of this patient population in general [35], emotional differences have been demonstrated between adolescent females and males [36]. Therefore, our study findings are limited to adolescent females with CMP and cannot be generalized to male patients. Future studies utilizing the freelisting method for this patient population should employ purposive sampling (based on biological sex, as well as race and ethnicity). Recruitment strategies to target and ensure inclusion of male (e.g., including male research staff to conduct the interviews) and non-English speaking subjects should also be employed.

Participant-centered language used to describe their pain condition included frustrating, upsetting, annoying, and painful. Participants identified family, friends, teachers, guidance counselors, and health professionals as key sources of support for their CMP and emphasized the utility in communicating their pain to these individuals, although many participants reported an absence of words used to communicate and explain their pain to others. While varied based on the setting, participants identified a portfolio of pain coping strategies, including rest, distraction, relaxation, endurance, and participation in extracurricular activities. Both positive and negative themes emerged from this study, offering critical insight into how to better support and learn from adolescents experiencing CMP.

Participants’ descriptions of positive relationships with their family, friends, teachers, guidance counselors, and health professionals codifies the importance of these roles in helping female adolescents with CMP. It is therefore key that medical and academic institutions, chronic pain organizations, health professionals, and communities support efforts to further foster these relationships and support pathways that increase exposure to helping systems. Formal programming to develop, strengthen, and maintain strength-based relationships should be central to CMP-directed care. Additionally, the relief and support that our interviewed adolescents felt from their doctors’ understanding and familiarity with CMP suggests the value of pain symptom validation by providers, potentially leading to increased trust, as another key piece of CMP-directed care.

Interestingly, many respondents reported that they do not talk to their family members about their pain, and some also avoid discussing it with their friends. Some participants also described difficulty explaining their condition to their teachers. On the one hand, the responses we recorded could reflect teens’ appropriate approach of avoiding discussion about their pain or attention to their pain, since our clinic routinely recommends that families refrain from talking about the adolescent’s pain. However, via probing questions, a subset of respondents offered some explanations for these communication difficulties, including a desire to avoid thinking about their pain, preference in coping alone, and the complexity of their condition. This suggests that CMP-directed care for females could explicitly emphasize improving communication strategies in a variety of young peoples’ relationships.

The invisibility of pain to others and the diagnostic uncertainty associated with CMP often engenders symptom disbelief and social rejection by others, leading to feelings of pain-related stigma among affected adolescents [37,38]. Emerging literature within the growing body of pain-related stigma research highlights the negative impact it has on pediatric pain populations and even describes it as an important social determinant of health among adolescents with pain conditions as well as a fundamental cause of health inequalities [39]. A recent study details the types of stigma (felt stigma, anticipated stigma and concealment, and internalized stigma) that adolescents with chronic pain experience from various social domains, including from medical providers, school personnel, family members, and peers [37]. Consequently, prior experiences with pain-related stigma often lead adolescents with CMP to conceal their pain symptoms as a coping strategy to avoid judgment, avoid feeling like a burden, and to feel more normal, which can reinforce social isolation and perpetuate negative school and health outcomes [37]. These concepts of pain-related stigma and concealment as coping strategies likely explain, at least partially, why our interviewed adolescents did not discuss or describe their pain to some of their close support networks. While our participants highlighted the value of social supports, their communication difficulties with these networks raises concern. Moreover, one tenant of trauma-informed care is the avoidance of re-traumatization. Further exploration of ways to bolster and expand effective communication related to chronic pain syndromes, mapped to adolescent-specific language choice, should be developed to provide appropriate orientation and feedback to strength-based supports, with the goal of reducing the burden placed on patients with CMP to explain their situations. It is critical that the CMP community develop interventions to address these issues of pain-related stigma and re-traumatization and their negative consequences [38].

In addition to the limited representativeness of this study sample, this study has some other limitations. First, the freelisting response is prone to recall bias, but the instructions to list items or words that come immediately to mind should have limited this bias. As described above, stigma may drive social desirability in shaping responses. While the freelisting exercise was able to generate a succinct list of responses, sometimes prompting questions were needed for further clarification; these varied by each interviewer–interviewee dyad which may have introduced some bias in the findings. The value of the lists without further explanation may also be questioned. Furthermore, there were no exclusion criteria for duration or frequency of follow-up care in clinic. This meant that there was variation among participants as to where they were in their treatment course. Finally, participants were recruited to participate in the freelisting interview within 6–8 weeks of their recent follow-up appointment. Since participants were selected based on follow-up appointments, those recruited could be more involved in their pain treatment or require more structure than other patients who do not return for follow-up.

## 5. Conclusions

Overall, this pilot study demonstrates the potential application of the freelisting method to succinctly ascertain qualitative data from adolescents with CMP–with a few disclaimers. First, the method may be more approachable for female participants, and mechanisms to ensure participation from male patients and delivery of freelisting to non-English speaking participants is paramount for future work to address diversity, equity, and inclusion in qualitative research. Nonetheless, our study demonstrates the willingness of female patients to participate in qualitative research efforts and the value we can gain by including the patient voice. The freelisting method is readily understood by female adolescents with chronic pain, allowing them to engage in this qualitative method to efficiently contribute data reflecting the patient perspective. We must continue to learn from patients’ lived experiences to further align their priorities with research priorities in the CMP field. By continuing to incorporate the patient perspective in CMP research endeavors, we can inform future approaches and interventions to more robustly study CMP and comprehensively care for this vulnerable patient population. Further refinement of the use of freelisting for this patient population could serve as a promising qualitative research method for achieving these goals.

## Figures and Tables

**Table 1:** Demographics and Clinical Characteristics of Final Study Participants (*n* = 27).

	*n* = 27
Female, *n* (%)	26 (96.3%)
Race	
Asian	1 (3.7%)
Black	4 (14.8%)
White	21 (77.8%)
Other	1 (3.7%)
Hispanic/Latino	3 (11.1%)
Age at time of initial diagnosis	14.7 (2.1)
Age at most recent clinic visit	16.0 (1.8)
Pain Subtype	
Diffuse pain	22 (81.5%)
Localized pain	5 (18.5%)
Resilience (CD-RISC-10 [0–40])	27.2 (6.4)
Pain intensity (Pain VAS [0–10])	5.8 (2.4)
Functional distability (FDI [0–60])	14.9 (9.2)
Pain widespreadness (WPI)	17.4 (29.8)
Somatization (SSS)	6.2 (2.8)
Fatigue (PROMIS Fatigue)	58.1 (12.1)
Peer relationships (PROMIS Peer Relationships)	50.4 (12.1)
Pain catastrophizing (PCS-C)	19.3 (10.1)

Legend. All measures are self-report. CD-RISC-10 = Connor-Davidson Resilience Scale 10-item. VAS = visual analogue scale. FDI = functional disability inventory. WPI = widespread pain index. SSS = symptom severity score. PROMIS = Patient reported outcome measure information system. PCS-C = pain catastrophizing scale child.

**Table 2:** Item List with Corresponding Salience Indices in Response to Question #1: When you’re alone and you feel pain, what do you do to feel better?

Item	Salience
rest	0.246
distract	0.225
relax	0.186
music	0.138
endure	0.133
unwell	0.103
read	0.094
exercise	0.085
desensitization	0.073
food	0.071
watch	0.067
people	0.061
stretch	0.06
digital	0.054
dog	0.047
art	0.038
write	0.038
video_games	0.038
isolate	0.038
nothing	0.038
games	0.038
focus	0.033
communicate	0.029
take_medications	0.023
dark	0.023
socialize	0.022
blankets	0.022
hydrate	0.019
walk	0.019
sad	0.019
tired	0.013
doing_something_boring	0.013
physical_pain	0.006

Legend. The term “rest” included all words related to ceasing movement to refresh or recover from pain, such as napping, sleeping, and laying down, among others. The term “distract” included all contractions of the word, such as distraction, as well as phrases related to diverting one’s attention, such as “entertain myself” and “redirect my mind”. The term “relax” included all words related to engaging in activities to become less anxious, such as deep breathing, meditate, and “calm down”. Activities involved in resting, relaxing, or distracting oneself were used to alleviate pain symptoms and feelings surrounding pain. One salient strategy identified included “music”, such as listening to music or playing an instrument. Participants who listed “endure” or similar phrases, such as “tough it out” and “push through”, described tolerating pain so that they can resume their activities and not miss out on being around others because of their pain.

**Table 3:** Freelisting Questions and Most Salient Items.

Question	Salient Terms (Salience Indices)
1. When you’re alone and you feel pain, what do you do to feel better?	Rest (0.246) Distract (0.225) Relax (0.186) Music (0.138) Endure (0.133)
2. What words come to mind when you think of or talk about your pain?	Frustrating (0.178) Sad (0.169)
3. When you’re with your family and you feel pain, what do you do to feel better?	Communicate (0.344) Distract (0.285)
4. What words do you and your family use to talk about your pain?	Not discussed (0.269)
5. When you’re with your friends and you feel pain, what do you do to feel better?	Communicate (0.283)
6. What words do you use when you’re with your friends to talk about your pain?	Not discussed (0.173) Medical condition (0.12) Annoying (0.096)
7. When you’re at school and feel pain, what do you do to feel better?	Focus (0.26) Distract (0.199) Leave (0.171) Relax (0.164) Endure (0.136)
8. What words do you use with your teachers to talk about your pain?	Painful (0.173)
9. What extracurricular or group activities are helpful for your pain?	Performance arts (0.332) Sports (0.249) Exercise (0.165) Visual arts (0.157)
10. What support networks are helpful for your pain?	Family (0.535) Friends (0.461)
11. What community members are helpful for your pain?	School community (0.307) Friends (0.293)
12. What health professionals are helpful for your pain?	Doctor (0.429) Physical therapist (0.309) Therapist (0.306)

## Data Availability

The data underlying this article cannot be shared publicly due to the privacy of individuals that participated in the study. The data presented in this study are available on request from the corresponding author.

## Supplementary Materials

The following supporting information can be downloaded at: https://www.mdpi.com/article/10.3390/children12010048/s1, The item lists and scree plots for freelisting questions #2–12 can be found in the supplementary material.
