# Supplementary material for: A Pilot Study on the Freelisting Method Among Adolescents with Chronic Musculoskeletal Pain: Feasibility, Acceptability and Study Findings
Source: Children (Basel). 2024 Dec 31;12(1):48. doi: 10.3390/children12010048 (PMC11763379; doi:10.3390/children12010048)

Question #02: What words come to mind when you think of or talk about your pain?

Item	Salience
frustrating	0.178
sad	0.169
uncomfortable	0.131
painful	0.124
hard_to_understand	0.111
annoying	0.1
exhausting	0.1
difficult	0.09
body_parts	0.083
constant	0.081
pain_descriptor	0.076
disbelief	0.07
relief	0.051
restrictive	0.038
medical_condition	0.038
medical_professional	0.038
break	0.038
medical_treatment	0.032
endure	0.026
distract	0.026
awful	0.023
coping_mechanisms	0.019
pain_is_distracting	0.019
sometimes_resourceful	0.019
not_discussed	0.015

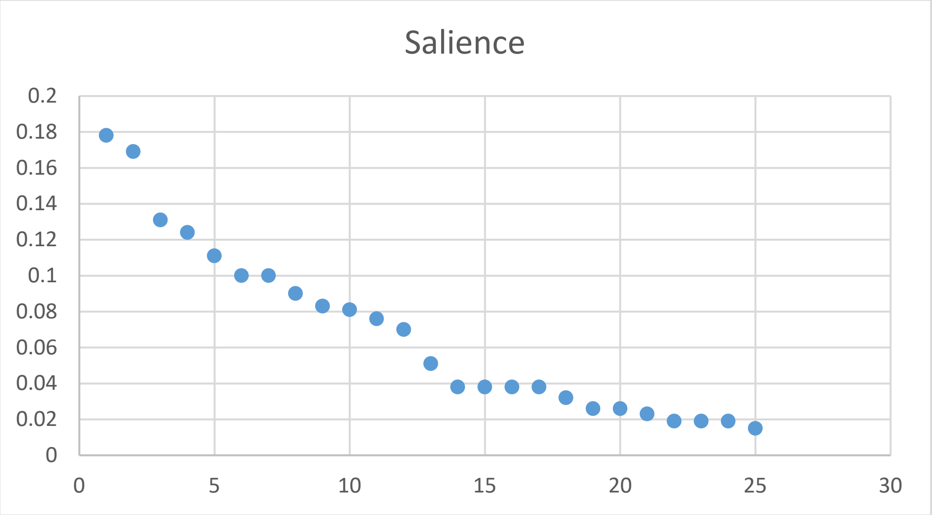

Question #03: When you're with your family and you feel pain, what do you do to feel better?

Item	Salience
communicate	0.344
distract	0.285
endure	0.192
isolate	0.154
games	0.127
avoid	0.125
nothing	0.115
rest	0.071
stretch	0.055
relax	0.045
sit	0.029
music	0.029
love	0.028
leave	0.026
food	0.026
walk	0.026
video_games	0.026
communicate-people	0.023
watch	0.023
desensitization	0.022
hydrate	0.019
write	0.019
focus	0.019
doing_something_boring	0.011

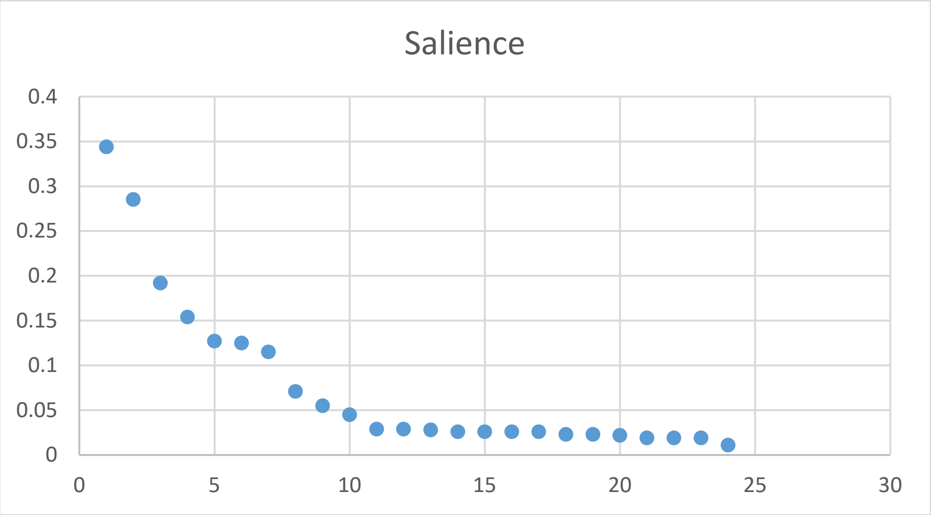

Question #04: What words do you and your family use to talk about your pain?

Item	Salience
not_discussed	0.269
hard_to_understand	0.087
pain_descriptor	0.08
painful	0.077
constant	0.077
fear	0.077
annoying	0.071
endure	0.067
difficult	0.067
pain_scale	0.058
frustrating	0.054
exhausting	0.048
uncomfortable	0.048
manageable	0.045
medical_condition	0.045
temporary	0.038
distanced	0.038
indirect	0.038
no_response	0.038
body_parts	0.038
discussed	0.031
debilitating	0.029
struggle	0.026
challenging	0.023
injury	0.019
journey	0.019
an_off_day	0.019
sad	0.019
disbelief	0.019
pain_is_distracting	0.019
coping_mechanism	0.016

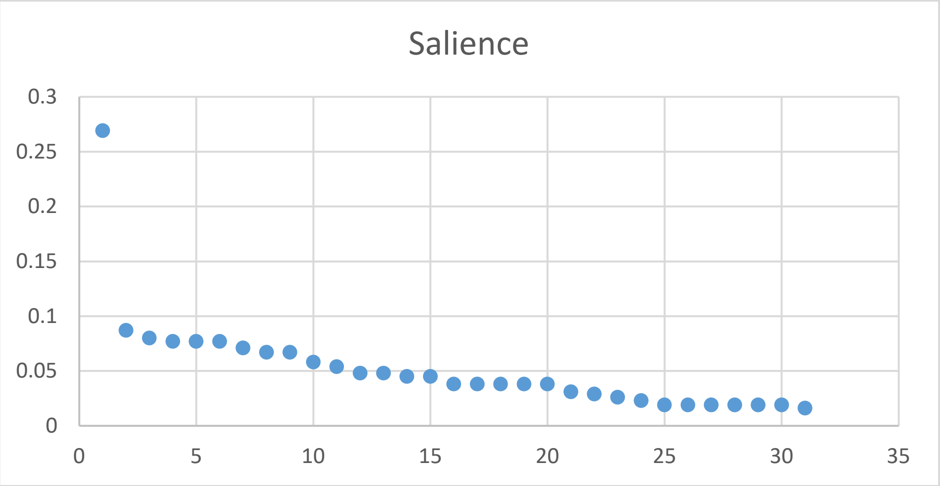

Question #05: When you're with your friends and you feel pain, what do you do to feel better?

Item	Salience
communicate	0.283
endure	0.123
nothing	0.111
humor	0.104
distract	0.102
relax	0.079
not_discussed	0.074
avoid	0.067
focus	0.065
stretch	0.056
video_games	0.054
rest	0.052
exercise	0.044
watch	0.04
no_pain	0.037
lacking_friendship	0.037
quizzes	0.037
acknowledged	0.037
music	0.037
sit	0.034
smile	0.032
activities	0.028
listen	0.026
leave	0.025
walk	0.022
openness	0.019
art	0.019
socialize	0.019
sing	0.015

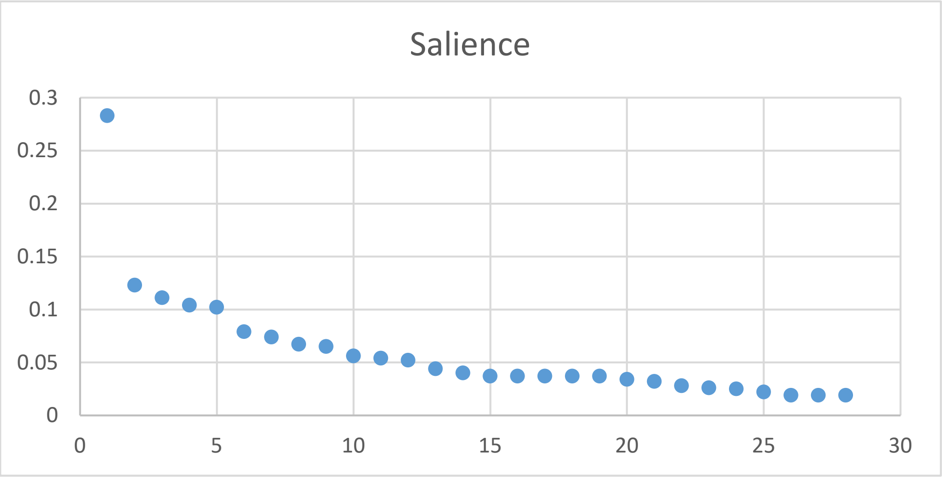

Question #06: What words do you use when you're with your friends to talk about your pain?

Item	Salience
not_discussed	0.173
medical_condition	0.12
annoying	0.096
pain_descriptor	0.08
exhausting	0.074
frustrating	0.067
body_part	0.06
sad	0.056
constant	0.056
painful	0.046
wheelchair	0.037
a_misfire_in_my_brain	0.037
distract	0.037
no_response	0.037
pain	0.03
manageable	0.03
difficult	0.028
hard_to_understand	0.028
pain_scale	0.028
discussed	0.025
awful	0.025
fear	0.025
dismiss	0.025
uncomfortable	0.022
setback	0.022
break	0.019
endure	0.015
obnoxious	0.015
product	0.015
pain_noises	0.012

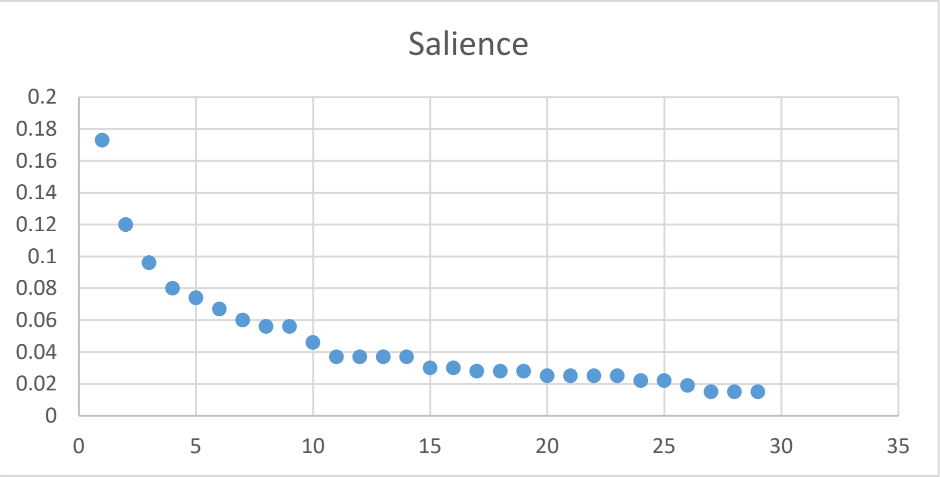

Question #07: When you're at school and feel pain, what do you do to feel better?

Item	Salience
focus	0.26
distract	0.199
leave	0.171
relax	0.164
endure	0.136
walk	0.11
nurse_office	0.099
communicate-people	0.096
nothing	0.074
stretch	0.056
read	0.056
write	0.043
communicate	0.041
avoid	0.037
absence	0.037
idk	0.037
music	0.037
segment	0.037
hydrate	0.034
listen	0.03
rest	0.025
managing	0.019
friends	0.012

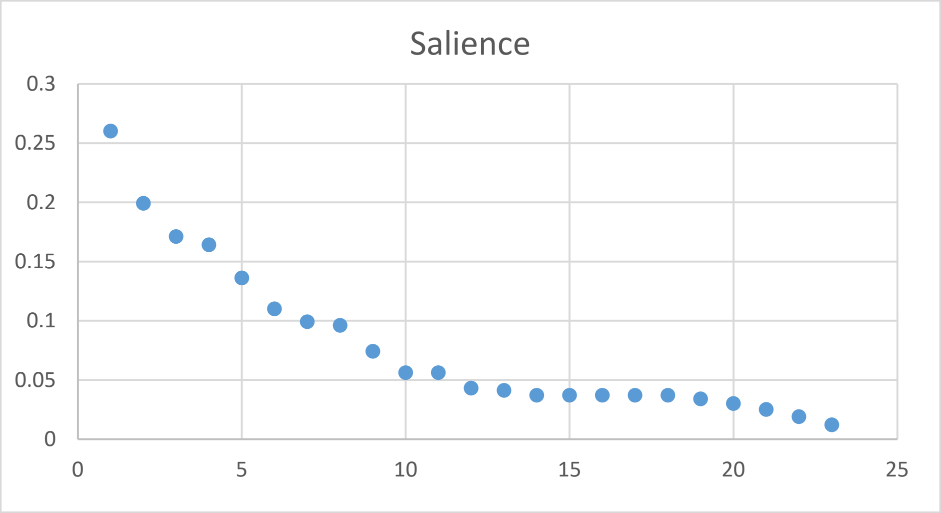

Question #08: What words do you use with your teachers to talk about your pain?

Item	Salience
painful	0.173
difficult	0.128
constant	0.114
no_response	0.111
frustrating	0.111
not_discussed	0.102
medical_condition	0.099
pain_is_distracting	0.083
hard_to_understand	0.068
exhausting	0.062
break	0.04
struggle	0.037
sorry_about_this	0.037
fear	0.037
pain_descriptor	0.032
medical_professional	0.032
walk	0.028
endure	0.025
capacity	0.025
uncomfortable	0.025
manageable	0.022
here_to_learn	0.019
debilitating	0.019
annoying	0.019
coping_mechanisms	0.019
different	0.015
body_part	0.015

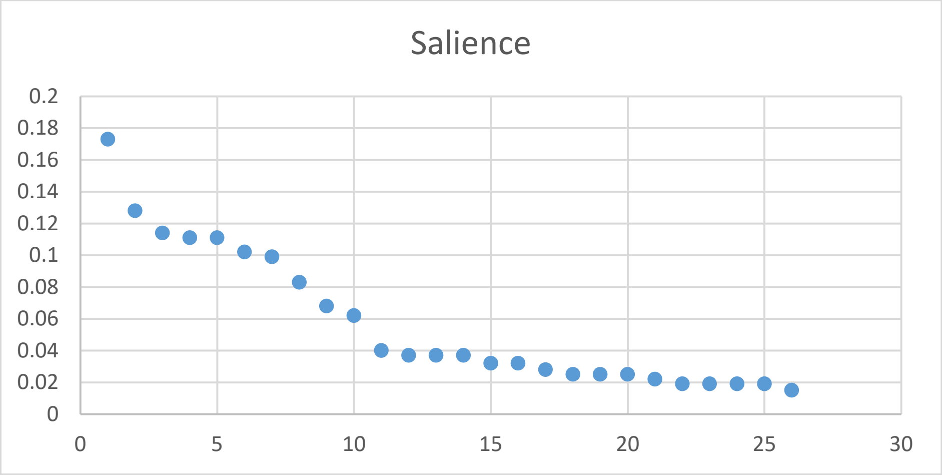

Question #09: What extracurricular or group activities are helpful for your pain?

Item	Salience
performance_arts	0.332
sports	0.249
exercise	0.165
visual_arts	0.157
club	0.095
walk	0.065
socialize	0.065
physical_therapy	0.062
activities	0.058
watch	0.047
low_impact	0.037
no_response	0.037
not_discussed	0.037
outdoors	0.037
rest	0.037
read	0.031
relax	0.026
stretch	0.025
everything	0.019
games	0.019
cooking	0.016
distract	0.015
dog	0.012

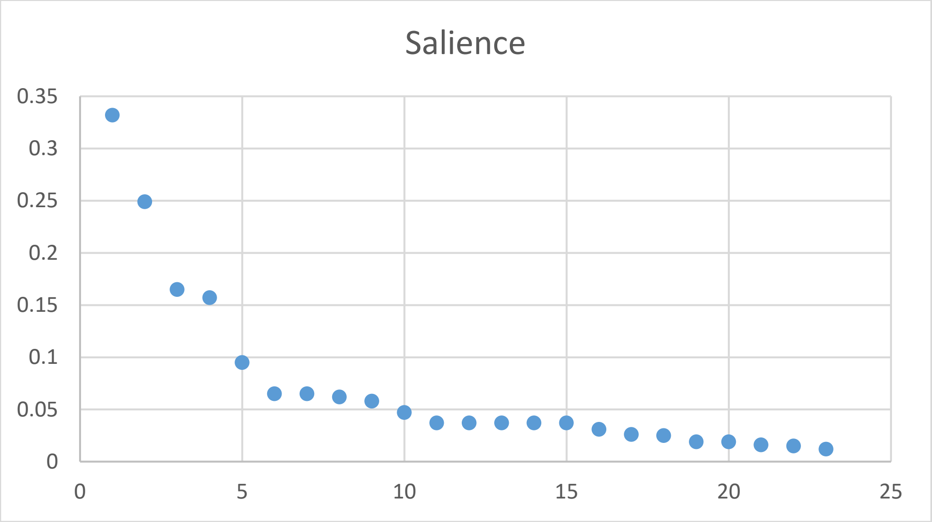

<u>Question #10: What support networks are helpful for your pain?</u>

Item	Salience
family	0.535
friends	0.461
therapist	0.175
school_community	0.152
online_community	0.114
medical_professional	0.101
no_response	0.074
watch	0.057
dog	0.042
desensitization	0.037
religion	0.037
trusted_adult	0.037
myself	0.025
roomate	0.023
exercise	0.022
read	0.019
sports_community	0.015

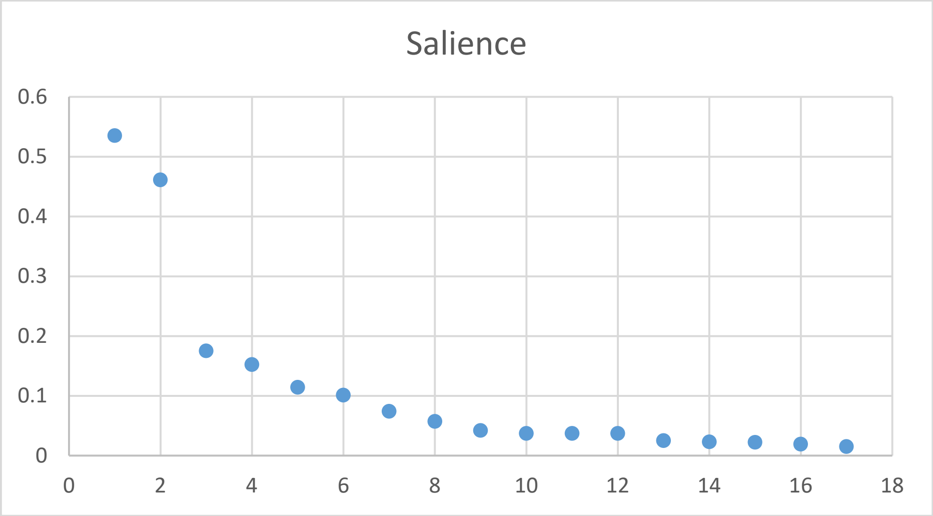

<u>Question #11: What community members are helpful for your pain?</u>

Item	Salience
school_community	0.307
friends	0.293
no_response	0.185
sports_community	0.14
myself	0.111
religion	0.09
family	0.077
neighbors	0.056
medical_professionals	0.049
everyone	0.037
therapist	0.025

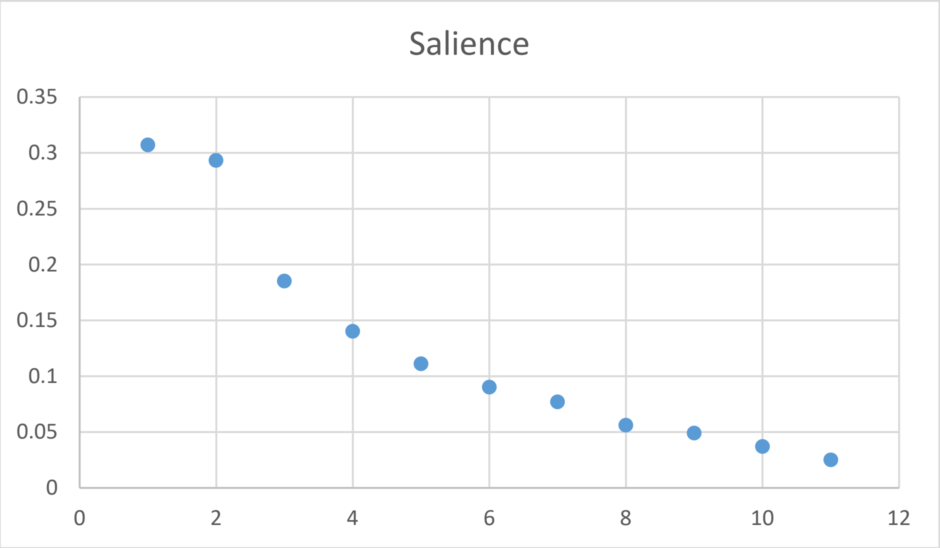

Question #12: What health professionals are helpful for your pain?

Item	Salience
doctor	0.429
physical_therapist	0.309
therapist	0.306
amps_provider	0.179
mentalhealth_provider	0.127
nurse	0.12
no_response	0.074
rheumatologist	0.056
trainer	0.043
pediatrician	0.043
neurologist	0.037
occupational_therapist	0.033
art_therapist	0.03
music_therapist	0.022
chiropractor	0.022
acupuncturist	0.015
orthopedist	0.012
orthopedist	0.012

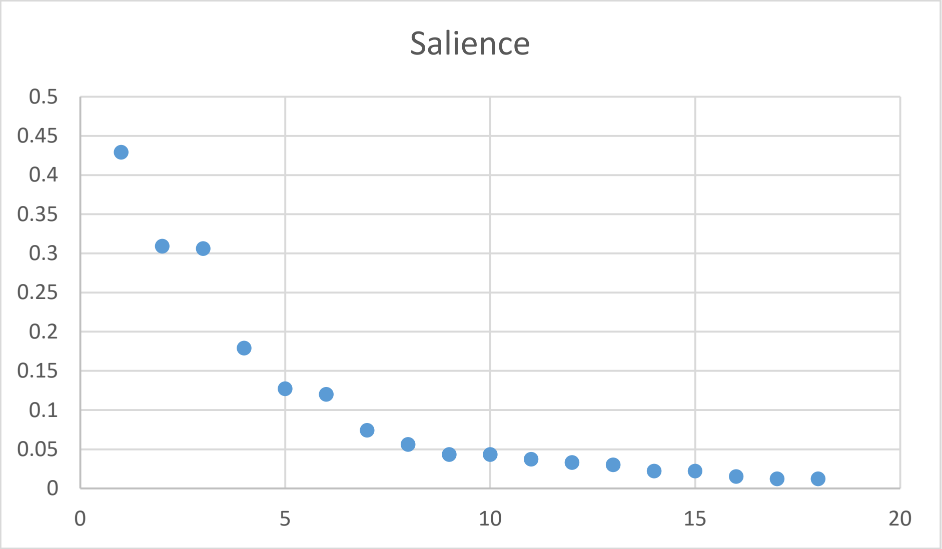

Supplement: Supplementary file 1 [file children-12-00048-s001.zip › children-3365983-supplementary.pdf]
